# Early post-operative anterior segment parameters modifications induced by PreserFlo MicroShunt in primary open-angle glaucoma

**DOI:** 10.1007/s10792-023-02697-z

**Published:** 2023-04-08

**Authors:** Gloria Gambini, Matteo Mario Carlà, Federico Giannuzzi, Francesco Boselli, Emanuele Crincoli, Tomaso Caporossi, Antonio Baldascino, Umberto De Vico, Alfonso Savastano, Stanislao Rizzo

**Affiliations:** 1 Ophthalmology Department, Fondazione Policlinico Universitario A. Gemelli, IRCCS, Largo A. Gemelli, 8, 00168 Rome, Italy; 2 Catholic University Sacro Cuore, Rome, Italy

**Keywords:** PreserFlo MicroShunt, Anterior segment parameters, Scheimpflug imaging, Central corneal thickness

## Abstract

**Purpose:**

The aim this study is to determine anterior chamber parameters variations induced by PreserFlo MicroShunt implantation, in the early post-operative days.

**Methods:**

This is a prospective observational study on 48 eyes undergoing PreserFlo MicroShunt implantation alone (*n* = 30) or combined with phacoemulsification (*n* = 18). Anterior chamber depth (ACD) and volume (ACV), central corneal thickness (CCT) and total corneal astigmatism (TCA) were evaluated pre-operatively, post-operatively at day-1 and at 1 week with the Pentacam tomography.

**Results:**

Intraocular pressure decreased significantly from 20.9 ± 4.0 to 8.0 ± 2.8 mmHg (*p* < 0.0001) and to 10.8 ± 3.7 mmHg (*p* = 0.0001) at day-1 and week-1, respectively. TCA varied significantly from baseline (1.5 ± 1.2 D) to both day 1 follow up (2.7 ± 1.9 D, *p* = 0.0003) and week 1 follow up (2.2 ± 1.6 D, *p* = 0.02). Nevertheless, only K1 showed a transient flattening at day 1, while K2 value didn’t show any statistical variation in the early post-operative period. CCT value rose significantly at day 1 (547 ± 49 vs. 529 ± 32 µm at baseline, *p* = 0.04), but then returned toward pre-operative values at week 1 (537 ± 39 µm, *p* = 0.57). In contrast, ACD values changed insignificantly from 3.3 ± 0.9 to 3.7 ± 1.0 mm at day 1 (*p* = 0.21), and then stabilized at 3.4 ± 0.9 mm (*p* = 0.82) at week 1 follow up. ACV changed from 150.0 ± 36.2 to 159.5 ± 42.1 mm^3^ at day 1 (*p* = 0.58), and successively to 153.9 ± 37.9 mm^3^ at week 1 follow up (*p* = 0.96). The subgroup analysis in eyes undergoing standalone PreserFlo implantation didn’t show significant changes in both ACD and ACV.

**Conclusion:**

PreserFlo implantation minimizes the anterior chamber modifications generated by traditional filtering surgery, inducing low and transient corneal and biometric changes only in the very early postoperative period and insignificant changes to ACD and ACV, label of its safety and minimal invasiveness.

## Introduction

Glaucoma filtering surgeries have the aim to bypass the trabecular meshwork by shunting aqueous humor (AH) to the subconjunctival or sub-Tenon space. [1] The gold standard trabeculectomy surgery has been shown to have effects on corneal biomechanical features, anterior segment parameters, axial length, and choroidal thickness. [2–6] The anterior chamber depth (ACD) after a filtering technique is well established to vary with axial length (AL), generally being shallower in the first days to weeks and progressively deepening as the post-operative time progresses. [7] According to some studies, it recovers to baseline during the second and third weeks following trabeculectomy, while, on the other hand, other researches found a reduction in the ACD at the conclusion of a 5-year follow-up period when compared to its baseline value. [8–10] Ocular biometric changes, such as corneal keratometric and topographical changes have been reported to follow also other glaucoma procedures, particularly non-penetrating deep sclerectomy and other bleb-forming procedures like the Ex-Press glaucoma implant (Alcon Laboratories Inc., Fort Worth, TX). [11–16] The latter showed a transitory influence on anterior segment parameters, such as ACD and anterior chamber volume (ACV), which appeared much lower on the first surgical day. [15]

MIGS (minimally invasive glaucoma surgery) refers to a series of relatively novel surgical techniques for lowering intraocular pressure (IOP), with the goal of a speedier recovery, decreased risk of complications, and less refractive and visual alterations than traditional trabeculectomy. [17] New ab interno and ab externo cylindrical implants have been created according to the Hagen-Poiseuille equation to restrict aqueous humor (AH) flow into the subconjunctival or sub-Tenon’s area in an attempt to decrease hypotony. [18]

The 8.5-mm PreserFlo MicroShunt (Santen Pharmaceutical Co., Osaka, Japan) is made of the stable and flexible polymer ‘SIBS’ (poly[styrene-blockisobutylene-block-styrene]), which showed its biocompatibility in cardiac stents. [19] It has a 70-µm lumen diameter and is placed ab externo through a 3-mm long scleral tract made using a 25G needle, with the aim to keep a steady aqueous outflow via the tube’s lumen to a posterior point at least 7 mm distant from the limbus, beneath Tenon’s capsule. [18] This procedure avoids the use of a scleral flap or sutures to control the volume and direction of the aqueous flow, with several recent reports gathering its effectiveness in IOP control. [20, 21] A recent report hypothesized that this sutureless and nonscleral flap-dependent approach, as well as deeper and more posterior filtering blebs, is able to determine less changes of anterior segment parameters, such as ACD and AL. [22]

The aim of our study is to determine anterior chamber (AC) variations, such as ACD, ACV, and corneal curvature parameters, induced by PreserFlo MicroShunt implantation, in the early post-operative days, to check whether the minimal invasiveness and reported safety of this device is also evident in the maintenance of anterior segment ocular parameters.

## Materials and methods

In this prospective observational study, we included patients with primary open-angle glaucoma (POAG) who underwent Microshunt PreserFlo implantation at Fondazione Policlinico Universitario A. Gemelli—Rome between November 27, 2019 and December 8, 2021. POAG was characterized by the presence of an open iridocorneal angle, evidence of glaucomatous optic nerve injury, and visual field (VF) deficits. The research comprised individuals who were on the most tolerated medication treatment and had increasing vision loss. This research adhered to the tenets of the Declaration of Helsinki and was approved by Catholic University of the Sacred Heart Ethical Committee in Rome, Italy. An informed and written consent was obtained from all enrolled patients.

We established the following inclusion criteria: uncontrolled glaucoma on maximum tolerated medication with an intraocular pressure (IOP) of 12–45 mmHg; best-corrected visual acuity (BCVA) of 20/200 or better; phakic or pseudophakic patients; and individuals with significant worsening of visual field parameters during a follow-up of at least 1 one year [mean deviation (MD), pattern standard deviation (PSD), visual function index (VFI), and glaucoma progression analysis (GPA)]. In the event of bilateral surgical indication, both eyes may be treated, if necessary, at least 1 month apart.

Exclusion criteria included prior filtering treatments (trabeculectomy), angle closure, pigmentary, pseudoexfoliative, uveitic, and neovascular glaucoma. In addition, the following exclusion criteria were considered: age less than 18, a history of intraocular inflammation or retinal abnormalities, a history of non-glaucomatous optic neuropathy, optic nerve drusen, poor image quality due to hypermature cataract, and unstable fixation. In situations with concomitant cataracts, implantation of PreserFlo together with phacoemulsification and IOL implantation was suggested. Consequently, our cohort was split in two subgroups: PreserFlo MicroShunt implantation alone or combined with phacoemulsification. All individuals provided written informed permission in accordance with the Declaration of Helsinki for this research. A total of 46 eyes who underwent PreserFlo implantation was included.

All patients’ demographics (age, sex, surgery date, laterality, and diagnosis), type of surgery (combined or stand-alone), and ocular parameters, were collected at the preoperative visit, including BCVA assessed with the Snellen chart, IOP at which the decision for surgery was made (measured with Goldmann applanation tonometer) and number of glaucoma medications at the preoperative visit. The post-operative IOP was analyzed at post-operative day 1 and week 1. At every follow-up, patients underwent three consecutive IOP measurements, 5 min apart from each other, and an average value from the three was collected. The number of patients with early adverse effects were also gathered.

### Surgical technique

All surgeries were performed by expert surgeons (S.R., T.C., A.S., L.M.) under peribulbar anesthesia. A traction suture on the superior cornea was used to expose the upper nasal conjunctiva in order to perform conjunctival peritomy and careful Tenon dissection, in order to create a posterior pocket in the supero-nasal quadrant. To reduce bleeding and provide a clean surgical area, a diathermy probe was used on the sclera.

All patients were treated with Mitomycin C (MMC) 0.2 mg/mL by placing three wet surgical sponges supplied by the manufacturer beneath Tenon’s layer for 2 min, avoiding limbus, and then abundantly washing with a balanced salt solution (BSS). With the tip of the caliper 3 mm away from the limbus, a trypan blue mark was made, and a microknife-made 1-mm wide scleral preincision was performede. A 25-G needle was inserted into the AC at the trabecular meshwork to form a scleral tunnel parallel to the sclera’s surface. The PreserFlo MicroShunt, just after priming with BSS to ensure its patency, was then put with the bevel up in the tunnel until reaching the AC, where it was visually verified to control that it was not too near to the iris or endothelium.

A planar attachment mechanism, like the fins of an arrow, is located halfway down the tube and secures the device in the pocket, avoiding tube migration. Flow through the implant was verified visualizing drop-by-drop flow from the end of the tube with a surgical sponge. Tenon’s layer was moved ahead of the conjunctiva to ensure that the implant did not become stuck in it, and the conjunctiva was then sutured watertight over Tenon’s layer using a 10–0 nylon fornix-based removable chain suture. [23] The surgical approach did not alter for combination surgery, and it was done at the conclusion of the phacoemulsification and IOL implantation process.

### Corneal tomography examinations

**Fig. 1:**
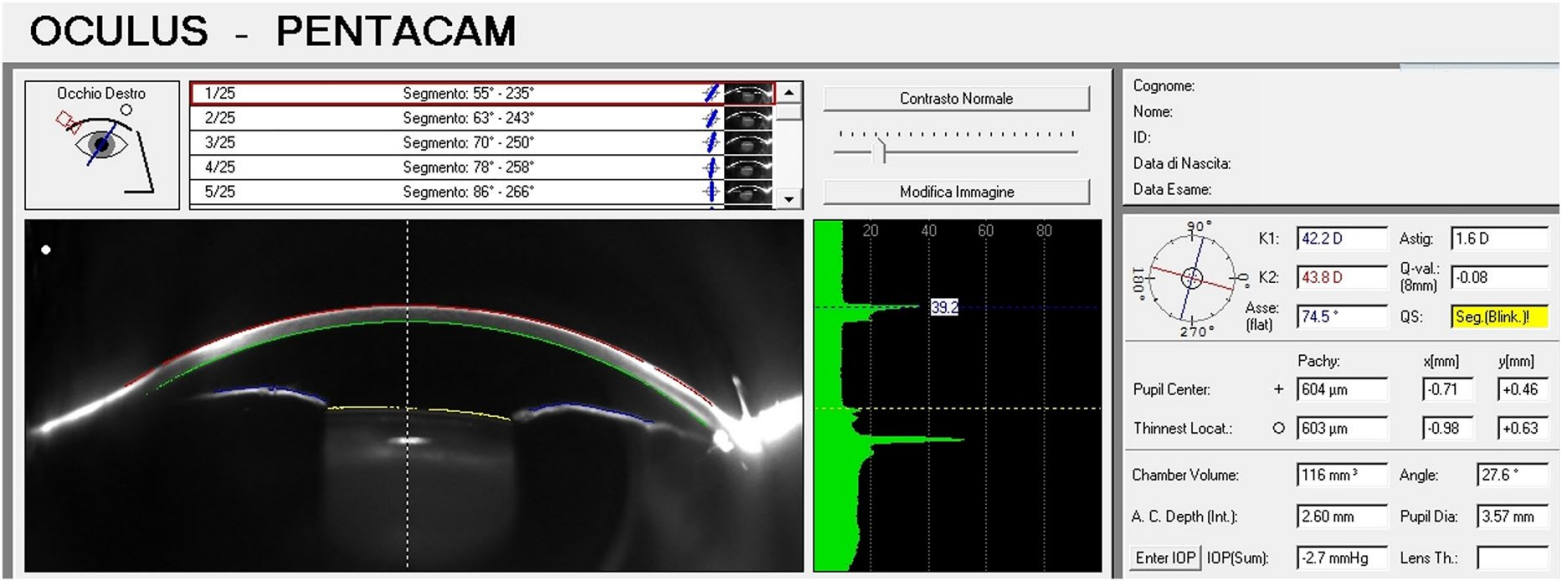
Scheimpflug image of the anterior segment showing the parameters taken in consideration in this study: corneal thickness at the pupil center (CCT), anterior chamber depth (ACD), anterior chamber volume (ACV), corneal curvatures parameters (K1, K2) and total corneal astigmatism (TCA)

Tomographic analysis were performed the day before surgery, and then repeated 24 h after surgical procedure and at the 7-days follow-up. The Pentacam HR (Oculus Inc.) is an eye tomography device exploiting a 360-degree rotating Scheimpflug camera and a monochromatic slit-light source (bluelight-emitting diode; wavelength, 475 nm) to obtain cross-sectional pictures of the anterior segment. This instrument can capture up to 50 photos in 2 s. Data regarding ACV, ACD, central corneal thickness (CCT), anterior corneal keratometries (Ks) and total corneal astigmatism (TCA) were collected (Fig. 1). The data were compared to a standard reference surface (best-fit-sphere) in the 8-mm zone which looked to be the most appropriate for clinical interpretation. [16]

### Statistical analysis

The statistical analysis was conducted using GraphPad PRISM Software, (Version 9.0; GraphPad, La Jolla, CA). Our sample’s normality was determined using the Shapiro–Wilk test, and *p* > 0.05 was utilized to confirm the null hypothesis. We performed Analysis of Variance (ANOVA) and Dunnett’s multiple comparison test with the Geisser–Greenhouse adjustment for matched pairs. To compare the difference between each pair of non-matched means, the Tukey test, which computes confidence intervals, was used. For contingency analysis, Chi-square and Fisher’s exact tests were utilized. In addition, correlation studies were performed on continuous variables. The quantitative results were represented as the mean standard deviation, and a *p* value 0.05 was deemed statistically significant. The 95 percent confidence interval (CI) of the mean was used.

## Results

A total of 48 eyes with POAG were included in the study. The study population’s mean age at the time of surgery was 72.2 ± 5.7 years; laterality (RE/ LE) was 30/18; sex (M/F) was 20/28. Mean preoperative IOP treated with anti-glaucoma drugs was 20.9 ± 4.0 (range, 13–28 mmHg), on a mean number of topical drugs of 2.8 ± 0.7; mean BCVA was 0.61 ± 0.3 and mean CCT was 529 ± 32 µm. A total of 28 patients (58%) were already pseudophakic, while 18 patients (37.5%) underwent combined surgery. Two eyes underwent standalone procedure without the need for cataract surgery. A total of 30 eyes underwent a standalone PreserFlo implantation (62.5%). Demographic charachteristics are visible in Table 1.

**Table 1:** Baseline demographic and clinical characteristics of the study population

	Eyes (*n* = 48)
Study eye, RE, no (%)	30 (63%)
Mean age, years	72.2 ± 5.7
Gender, male, no (%)	20 (42%)
BCVA ± SD, decimals	0.61 ± 0.3
Mean IOP ± SD; mmHg	22.0 ± 3.3
Mean CCT ± SD, µm	529 ± 32
Mean no. of IOP-lowering drugs ± SD	2.8 ± 0.7
History of phacoemulsification (%)	28 (58%)
Combined surgery (%)	18 (37.5%)

RE —right eye; BCVA —Best Corrected Visual Acuity; IOP— intraocular pressure; CCT— central corneal thickness; SLT— selective laser trabeculoplasty

PreserFlo MicroShunt decreased IOP from a mean baseline of 20.9 ± 4.0 to 8.0 ± 2.8 mmHg (*p* < 0.0001) at the first post-operative visit after 24 h, and to 10.8 ± 3.7 mmHg at 1 week (*p* = 0.0001).

In accordance with the World Glaucoma Association’s guidelines, hypotony was defined as an intraocular pressure (IOP) of 5 mmHg following surgery. [24] The incidence of hypotony in this series was 19% (*n* = 9) at day 1 and 8% (*n* = 4) at week 1, but resolved in all instances at the successive follow ups. In the first week, one of the patients developed choroidal effusion, which was successfully managed with conservative therapy (occlusion and 1% atropine eye drops), and resolved within the first month. Early self-limiting hyphema was often documented (*n* = 8, 17%) but fully resolved at week 1 in all instances.

### Tomographical changes

The findings of the change in anterior chamber parameters as well as anterior astigmatism are shown in Table 2.

**Table 2:** Comparison of pre-operative and post-operative parameters

Parameter (mean ± SD)	Pre-operative	Post-operative (1 day)		Post-operative (1 week)	
			*p*		*p*
IOP, mmHg	22.0 ± 3.3	8.0 ± 2.8	< 0.0001*	10.8 ± 3.7	0.0001*
*Pentacam*					
Anterior K1, D	43.8 ± 1.6	42.5 ± 1.7	0.005*	43.1 ± 1.8	0.19
Anterior K2, D	45.2 ± 2.0	45.3 ± 1.9	0.99	45.2 ± 1.9	0.99
Mean K, D	44.5 ± 1.7	43.9 ± 1.5	0.16	44.3 ± 1.7	0.38
TCA	1.4 ± 1.2	2.7 ± 1.9	0.0003*	2.1 ± 1.6	0.02*
ACD, mm	3.3 ± 0.9	3.7 ± 1.0	0.21	3.4 ± 0.9	0.82
ACV, mm^3^	150.0 ± 36.2	159.5 ± 42.1	0.58	153.9 ± 37.9	0.96
CCT, µm	529 ± 32	547 ± 49	0.04*	537 ± 39	0.57

*K*—keratometry, *IOP*—intraocular pressure, *TCA*—anterior corneal astigmatism, *ACD*—anterior chamber depth, ACV—anterior chamber volume, *CCT,*—central corneal thickness

Compared to the pre-operative period, we found a statistical difference (*p* = 0.005) in corneal K1 at day 1, from 43.8 ± 1.6 D (CI 43.3–44.2) to 42.5 ± 1.7 D (CI 42.0–43.0), which was not significant at week 1 (43.1 ± 1.8 D, CI 42.6–43.7, *p* = 0.19). On the other hand, no statistical variations were found in corneal K2, neither at day 1 (45.3 ± 1.9 D, CI 44.7–45.8, *p* = 0.99), nor at week 1 (45.2 ± 1.9 D, CI 44.6–45.8, *p* = 0.99). Consequently, a statistical difference was found when comparing TCA from baseline (1.5 ± 1.2 D) with both day 1 follow up (2.7 ± 1.9 D, *p* = 0.0003) and week 1 follow up (2.2 ± 1.6 D, *p* = 0.02). Nevertheless, mean *K* value didn’t show any statistical variation in the early post-operative period (from 44.5 ± 1.7 D, CI 43.9–45.0; to 43.9 ± 1.5 D at day 1, CI 43.4–44.3, *p* = 0.16; and 44.3 ± 1.7 D at week 1, CI 43.8–44.7, *p* = 0.38, respectively).

**Fig. 2:**
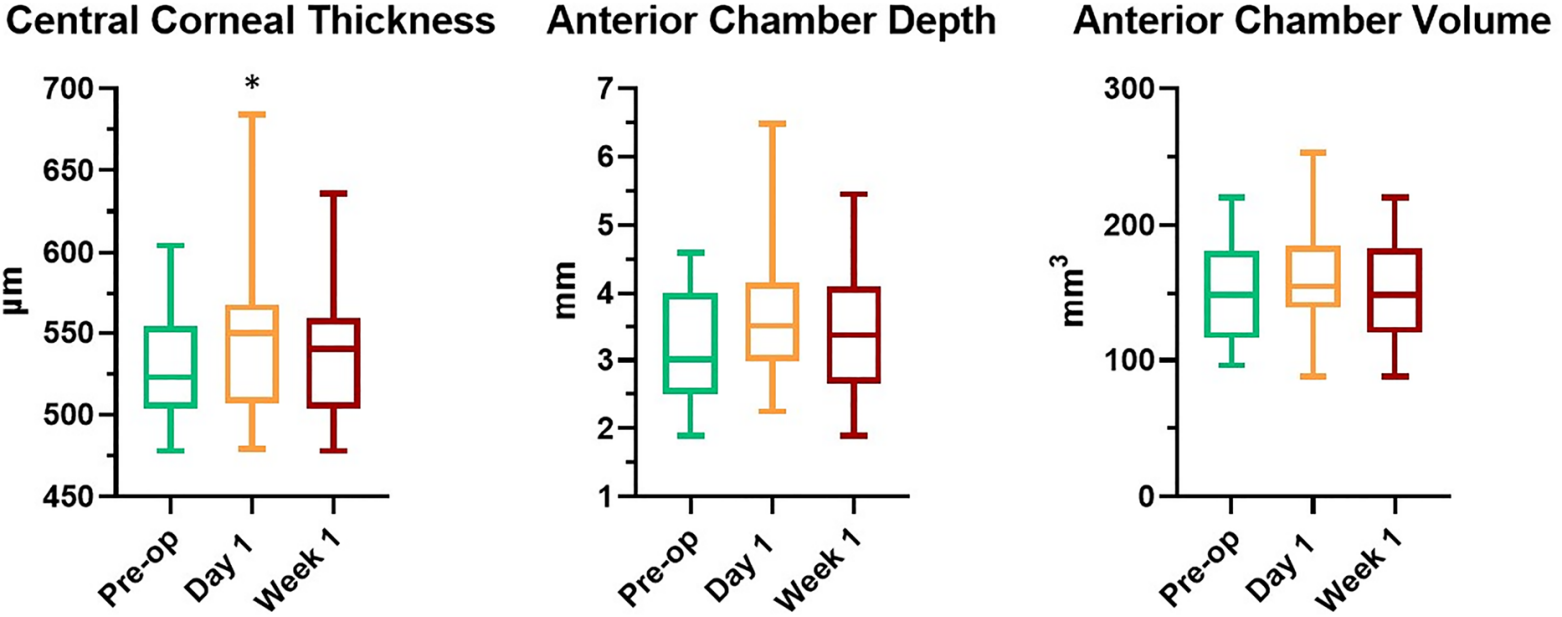
Box and plot diagrams of central corneal thickness (CCT), anterior chamber depth (ACD) and anterior chamber volume (ACV) variations from baseline to day-1 and week-1 follow ups

In the first postoperative week, CCT value rose significantly at day 1 when compared to baseline values (529 ± 32 µm, CI 520–538 vs. 547 ± 49 µm, CI 532–561, *p* = 0.04), but then returned toward pre-operative values at week 1 (537 ± 39 µm, CI 525 to 548, *p* = 0.57). In contrast, ACD and ACV didn’t show any significant variation at both follow ups. ACD values changed from 3.3 ± 0.9 mm, CI 2.9–3.5, to 3.7 ± 1.0, CI 3.3–4.0 at day 1 (*p* = 0.21), and then stabilized at 3.4 ± 0.9, CI 3.1–3.7 (*p* = 0.82) at week 1 follow up. ACV changed from 150.0 ± 36.2 mm^3^, CI 139.2–160.7, to 159.5 ± 42.1 mm^3^, CI 146.7–172.4, at day 1 (*p* = 0.58), and successively to 153.9 ± 37.9 mm^3^, CI 142.5 to 165.2, at week 1 follow up (*p* = 0.96) (Fig. 2).

A subgroup analysis was conducted in order to exclude the effect of lens extraction as a bias for ACV and ACD changes after surgery. In particular, ANOVA tests were applied specifically to the standalone PreserFlo surgery group (*n* = 30). As a result, ACD values didn’t show significant variations when comparing pre-operative values (3.1 ± 0.7 mm) to day-1 (2.9 ± 1.0 mm, *p* = 0.26) and week-1 (3.0 ± 0.9 mm, *p* = 0.46) follow ups, respectively. Similarly, ACV remained statistically stable when comparing baseline values (155.0 ± 24.2 mm^3^) to post-operative day 1 (143.5 ± 44.3 mm^3^, *p* = 0.22) ad week 1 (149.9 ± 33.5 mm^3^, *p* = 0.39).

When comparing post-operative TCA variations between standalone PreserFlo surgery and combined surgery groups, we found higher astigmatism changes in the latter (1.0 D vs. 1.3 D, *p* = 0.02) at day 1, which decreased towards the first week (0.6 D vs 1.0 D, *p* = 0.003). In particular, we found that the group of combined surgery showed a significant flattening in K1, which was linked to the site of the 2.2 mm clear corneal incision, at both day 1 (43.7 ± 1.9 D vs. 42.1 ± 1.4 D, *p* = 0.002) and day 7 (43.7 ± 1.9 D vs. 42.8 ± 1.5 D, *p* = 0.03). On the other side, K2 showed no significant changes (*p* = 0.36). In the standalone group, we reported no significant variation in both K1 and K2 (*p* = 0.15 and *p* = 0.48, respectively).

Moreover, the correlation analysis between IOP variation and the changes in all study variables (mean corneal K, TCA, ACD, ACV and CCT) revealed no statistically significant association, as visible in Table 3.

**Table 3:** Correlation between IOP changes and anterior segment parameters variations from baseline to day 7

	Correlation coefficient	*p*
ΔIOP versus Δmean K	0.21	0.18
ΔIOP versus ΔTCA	0.38	0.07
ΔIOP versus ΔACD	− 0.16	0.48
ΔIOP versus ΔACV	0.08	0.58
ΔIOP versus ΔCCT	− 0.15	0.10

Δ,—difference between baseline and last follow-up; *K*—keratometry, *IOP*—intraocular pressure, *TCA*—anterior corneal astigmatism, *ACD*—anterior chamber depth, *ACV*—anterior chamber volume, *CCT*—central corneal thickness

## Discussion

Previous studies reported that trabeculectomy caused considerable alterations in anterior segment parameters in the first postoperative week, which generally reverted to baseline in the first postoperative month. [25, 26] In fact, the rapid reduction in IOP induced by trabeculectomy may influence anterior segment characteristics in the early postoperative phase. [3]

Moreover, a widespread interest was focused on the impact of glaucoma surgery on astigmatism. Initially, Hugkulstone, using keratometry, observed a statistically significant decrease in vertical corneal radius from 7.71 mm preoperatively to 7.36 mm on day 1 postoperatively, which persisted at all time periods up to 28-days follow-up. [11] Claridge et al. described topographical complex regional changes in the corneal curvature that were not detected by changes in refractive or keratometric parameters and lasted for at least 12 months after trabeculectomy. [12] Cunliffe et al. similarly reported an 8-weeks lasting decrease in vertical corneal radius, which induced a with-the-rule (WTR) astigmatism, but reverted to preoperative values at 10 months. He hypothesized that the sclerostomy used in trabeculectomy causes the cornea’s edge to retract, resulting in a smaller vertical corneal radius. [9]

Overtight scleral flap sutures around 12 o’clock, according to Dietze et al., might induce local tissue compression, steepening the vertical meridian. [14] Astigmatism induction has also been linked to large drainage blebs, hypotony and to the use of mitomycin C, which seems to be linked to longer-lasting astigmatic alterations. [27] After trabeculectomy with MMC 0.4 mg/mL, Kook et al. identified a WTR change of 1.23 D at 3 months, which was sustained at 6–12 months (0.94 and 0.65 D). [6] Delbeke et al., on the other hand, found that 0.2 mg/ mL MMC resulted in a lesser WTR change (0.35D at 1 months, 0.18 D at 3 months follow up). [28]

Tzu et al. assessed the refractive results of cataract-only and combined cataract with trabeculectomy (using 0.4 mg/mL MMC) or Ahmed (New World Medical Inc., California) or Baerveldt (Abbott Laboratories Inc., Illinois) implants, reporting a difference in cylinder of 1.31 ± 0.86 in the combination group against 0.99 ± 0.72 in the cataract-only group, which was not statistically significant. Moreover, no difference between the combined trabeculectomy and the glaucoma draining devices (GDD) groups was found. [29]

Ioannidis et al. investigated refraction, visual acuity, and manifest astigmatism, using a vector-based analysis, in 106 eyes undergoing combined femtosecond laser-assisted cataract surgery and the implantation of two iStent inject trabecular microbypass devices (Glaukos, San Clemente, CA). As a result, the mean cylinder dropped from 0.91 D preoperatively to 0.41 D after 4 weeks, with 73.8% of eyes having 0.5 D or less residual refractive astigmatism following the procedure. [30] Hammel et al., in a Scheimpflug analysis, reported a transient increase in both anterior (2.6–4.7 D) and posterior (0.4–0.9 D) corneal astigmatism, which was higher than the increase seen in our series with PreserFlo MicroShunt. [15]

The use of Pentacam Scheimpflug tomography helped us to reduce possible corneal astigmatism underestimation, given its high accuracy in analyzing anterior and posterior corneal surfaces. [31] A recent research conducted by Ibarz Barberà et al. used the Pentacam system to measure corneal astigmatism, finding a 0.8 D change in TCA in the very early postoperative period after PreserFlo MicroShunt implantation, which decreased up to 3 months postoperatively, with a residual difference of 0.4D from baseline. [22] These results were consistent with our research, in which a 1.3 D change in TCA was found 24 h after surgery, but rapidly decreasing to 0.7 D at the 1-week follow up. When comparing post-operative TCA variations between standalone PreserFlo surgery and combined surgery groups, we found higher astigmatism changes in the combined group (1.0 D vs. 1.3 D) at day 1, decreasing towards the first week (0.6 D and 1.0 D in the two subgroups, respectively). Similarly, Ibarz Barberà et al. found more alterations in the combined phaco-PreserFlo MicroShunt subgroup (1 D rise in anterior surface astigmatism and 1.2 D increase in TCA), which faded by the third month, leaving a residual difference from baseline of 0.4 D of TCA in the non-combined group and 0.2 D in the combined group. [22] In our research, we found that corneal curvature changes were significantly linked to phacoemulsification procedure rather than PreserFlo implantation, since only the combined group showed a variation in K1 (from 43.7 ± 1.9 D to 42.1 ± 1.4 D, *p* = 0.002).

The advantage of the PreserFlo MicroShunt implant relies on the fact that scleral flap and sutures are not needed, eliminating two of the risk factors for the induction of astigmatism. Furthermore, some recent anterior segment optical coherence tomography (AS-OCT) analysis showed that the most common filtering bleb morphologies determined by PreserFlo MicroShunt are the microcystic multiform and the multiple internal layer ones, located posteriorly in relation to the limbus, with no cases of large or avascular blebs linked to changes in corneal curvature reported to date. [32, 33] To the best of our knowledge, no studies have been published that detail refractive alterations and induced astigmatism that occur after other MIGS implantation, such as the XEN gel stent.

On the other side, several works have neen published regarding the variation of ACD after glaucoma surgery. The first investigations were conducted with the Haag–Streit pachymetry, indicating that ACD achieves its shortest value on the second and third days after trabeculectomy, then deepens and reaches its preoperative value by the 14th day following surgery, as reported by Cunliffe et al. [9] Husain et al., using A-scan ultrasonography, found that the ACD value following trabeculectomy was considerably lower than the baseline value, and that this difference was maintained even 5 years later. [10]

The introduction of Scheimpflug imaging offered novel results in this setting, with a recent research showing how ACD levels rose from the first postoperative week to the first postoperative month in this research, with no significant difference between preoperative and first postoperative-month ACD values. In the same research, TCA and ACV values fell in the same way as the ACD value in the first postoperative week, but showed no statistical difference after 1 month when compared to the baseline values. [26] Similarly, Karasheva et al. used the IOLMaster, a noncontact device, to evaluate ACD in 44 trabeculectomized eyes, and found no significant change between pre- and post-surgical ACD readings. [34]

Previous research on the influence of trabeculectomy on CCT levels has shown mixed findings, with Moschos et al. showing a slow but substantial decline in these values in the first, sixth, and 12 months following trabeculectomy as compared to preoperative values, [35] while Huang et al. claimed that CCT values rose on the first day after trabeculectomy and reduced to baseline levels in the first week, similar to what happened in our research after PreserFlo MicroShunt implantation (from 529 µm at baseline to 547 µm at day 1, then decreasing to 537 µm at day 7). [36] Early postoperative increases in CCT values may be associated with surgically induced or hypotony related corneal edema, and postoperative decreases in CCT values may be associated with lower IOP and improvement in surgically induced corneal stress and endothelial function. [26]

More studies were conducted focusing on anterior chamber parameters variations after MIGS surgery. Hammel et al. reported the first Pentacam Scheimpflug analysis after Ex-PRESS Miniature Glaucoma Implant surgery in 2013, showing that surgery had a temporary influence on anterior segment characteristics such as posterior corneal astigmatism, ACD, and ACV. [15] Differently from our research, on the first surgical day, ACV and ACD were significantly reduced, and stabilized from the 1-week visit to the 3-month follow-up. Moreover the authors reported a correlation between IOP variations and corneal alterations, however this was not supported by our research.

Based on our findings, we hypothesize that the PreserFlo MicroShunt structure is able to keep a steady aqueous outflow via the tube’s lumen even from the very first hours after surgery, since we found a non-significant variation in both ACD (3.3 mm preoperatively vs. 3.7 mm the day after surgery) and ACV (150.0 mm^3^ preoperatively vs. 159.5 mm^3^ at day 1), allowing for more reproducible surgical results and increased safety. These results were comparable in both the standalone and the phaco-combined surgical approach and appeared to stabilize early, with the 1-week follow up parameters reported to be similar to baseline. As far as we know, this is the first research to analyze ACV variations after PreserFlo MicroShunt implantation, confirming the minimal invasiveness of this approach which consistently reduced the risk of shallow anterior chamber in the first post-operative days.

This research has several limitations, primarily due to the short-term follow up period, but has an higher sample size (*n* = 48) than other researches in this field, allowing for a better statistical reliability. In conclusion, the PreserFlo MicroShunt approach seems to restrain the anterior chamber modifications generated by traditional filtering surgery. In comparison to trabeculectomy, which has been reported to cause visually significant changes in the ACD, ACV and keratometric parameters that can last for more than a year in some cases, our research suggest that the implantation of this device determines slight modifications to ocular biometrics, inducing low and transient corneal and biometric changes only in the very early postoperative period and, even more important, insignificant changes to ACD and ACV, which is a label of its safety and minimal invasiveness. More research regarding AC changes, also combined with AS-OCT analysis, as well as a longer follow-up time, are certainly required to understand the long-term effects of the PreserFlo MicroShunt implantation on the anterior segment parameters.

## Data Availability

The data that support the findings of this study are available from the corresponding author, MMC, upon reasonable request.
